# Determining prognostic factors and optimal surgical intervention for early-onset triple-negative breast cancer

**DOI:** 10.3389/fonc.2022.910765

**Published:** 2022-10-28

**Authors:** Yi-Zi Zheng, Yan Liu, Zhen-Han Deng, Guo-Wen Liu, Ni Xie

**Affiliations:** ^1^ Department of Thyroid and Breast Surgery, Shenzhen Breast Tumor Research Center for Diagnosis and Treatment, The First Affiliated Hospital of Shenzhen University, Shenzhen Second People’s Hospital, Shenzhen, Guangdong, China; ^2^ Department of Critical Care Medicine and Infection Prevention and Control, the First Affiliated Hospital of Shenzhen University, Shenzhen Second People’s Hospital, Shenzhen, Guangdong, China; ^3^ Department of Sports Medicine, the First Affiliated Hospital of Shenzhen University, Shenzhen Second People’s Hospital, Shenzhen, Guangdong, China; ^4^ Biobank, First Affiliated Hospital of Shenzhen University, Shenzhen Second People’s Hospital, Shenzhen, Guangdong, China

**Keywords:** triple-negative breast cancer (TNBC), early-onset breast cancer, surgical intervention, prognosis, nomogram, propensity score matching (PSM)

## Abstract

**Background:**

Few studies have focused specifically on prognostic factors and optimal surgical intervention for early-onset triple-negative breast cancer (eTNBC), which is characterized by high malignancy and poor prognosis.

**Methods:**

We performed a cohort study with a median follow-up of 31 months using Surveillance, Epidemiology, and End Results (SEER) data of patients diagnosed with stages I–III eTNBC between 2010 and 2016. In addition, we collected cases between 2006 and 2016 from our center as an external validation set. Clinical features, pathologic characteristics and oncologic outcomes were analyzed. Prognostic factors for overall survival (OS) and breast cancer-specific survival (BCSS) were determined by Cox proportional hazards analyses and were incorporated into the prognostic nomogram. Subgroup analysis based on propensity score matching method was conducted to explore the subset of patients that would benefit from breast-conserving therapy (BCT).

**Results:**

Based on SEER dataset, patients with eTNBC were more likely to undergo mastectomy than BCT. On multivariable analysis, patients with better survival outcomes were those not married, uninsured, had higher T and N stage, and had histological type of mixed invasive ductal and lobular carcinoma. The prognostic nomogram based on these variables successfully predicted the 3- and 5-year BCSS (C-index in training cohort, 0.774; in validation cohort from SEER, 0.768; in validation cohort from our center, 0.723). Subgroup analysis illustrated that patients with T1N0M0 or T2-4N+M0 tumors who underwent BCT achieved longer overall survival than those who underwent mastectomy (for T1N0M0, *P* = 0.022; for T2-4N+M0, *P* = 0.003); however, the type of surgery did not influence OS among patients with T1N+M0 or T2-4N0M0 tumors (for T1N+M0, *P* = 0.305; for T2-4N0M0, *P* = 0.317).

**Conclusions:**

The prognosis of patients with eTNBC is mainly affected by marital status, insurance status, T stage, N stage and histological type. The prognostic nomogram based on these factors is quite reliable. Subgroup analysis suggested that BCT may be a superior option for patients with eTNBC, especially those with T1N0M0 and T2-4N+M0 tumors.

## Introduction

Breast cancer is the most common malignancy among women worldwide. In the United States alone, 281,550 women were newly diagnosed with the disease in 2021 and 43,600 died of it (1). Among them, the incidence rate of early-onset breast cancer has increased significantly since 2000 (2). Accumulating evidence suggests that young age breast cancer is the leading cause of cancer-related deaths of women under the age of 45 years (3). Early-onset breast cancer is defined as a breast malignancy diagnosed in a patient who is under 40 years of age; while accounting for 5.6–6.6% of breast cancers, it is responsible for a disproportionately higher number of disease-related deaths (4, 5). Patients with early-onset breast cancer reportedly present with more aggressive disease than older counterparts and undergo chemotherapy and mastectomy at higher rates (6, 7). Their clinical outcomes are worse, while the biology and epidemiology of their breast tumors are distinct (5, 8).

Four surrogate molecular subtypes of breast cancer have been identified to date: luminal A (estrogen receptor [ER]-positive, progesterone receptor [PR]-positive, human epidermal growth factor receptor 2 [HER2]-negative, and a low Ki67 index [Ki67-low]), luminal B (ER-positive, PR-negative or Ki67-high, and either HER2-positive or HER2-negative), triple negative breast cancer (TNBC) (ER-negative, PR-negative, and HER2-negative), and HER2-overexpressing tumors (9). Among these, TNBC remains a heterogeneous subset of breast cancer that tends to behave more aggressively and has a poorer prognosis (10). Importantly, TNBC is overrepresented in patients with early-onset breast cancer, afflicting 26% of this population compared to 12% overall (11, 12). There is marked heterogeneity in the outcomes of young patients with TNBC owing to individual characteristics, tumor burdens, and the underlying tumor biology (13, 14). Therefore, it is of great significance to evaluate the prognosis of patients with early-onset triple-negative breast cancer (eTNBC). Besides, breast loss has a great impact on marital relationship, social life and workplace mentality, and young patients are often more willing to receive breast-conserving therapy (BCT, breast-conserving surgery combined with radiotherapy) than elder patients do (15). It is therefore important to determine whether BCT is safe in eTNBC.

Although most studies of TNBC have focused on the evolution of systemic treatments, less attention has been paid to oncological outcomes in relation to locoregional treatment. Several studies have demonstrated that patients with TNBC and HER2-overexpressing subtypes of breast cancer who undergo BCT have higher risks of both locoregional recurrence and distant metastasis than do those with luminal subtypes (16–18). Such findings had raised concerns regarding the safety of BCT for patients with TNBC. Other studies have suggested that receiving BCT does not lead to a higher local recurrence rate of TNBC, instead, the worse overall survival of patients is due to triple-negative subtyping itself (19–24). It was even found that BCT had a survival benefit for TNBC compared with total mastectomy (25). This might be due to the fact that most reviews and observational studies focused on the prognostic impact of BCT for TNBC versus other molecular subtypes of breast cancer, and therefore only included patients who received BCT (2, 20, 26, 27); whereas other studies looked at the effect of different surgical interventions on prognosis and included patients who had undergone total mastectomy (18, 25). Therefore, it is the current consensus that TNBC is not contraindicated to BCT (21). In addition, limited studies suggested that although young age is an independent prognostic factor for an increased rate of local recurrence after BCT (16), young patients received BCT or total mastectomy had similar overall survival (28). It warrents further study that whether BCT in patients with eTNBC leads to survival outcomes similar to or even better than total mastectomy.

Owing to the limited number of subjects, there is currently few research focusing on optimal surgical intervention for patients with eTNBC. We conducted this population-based study of eTNBC in an attempt to identify prognostic factors and the subpopulation who would benefit from BCT. Previous studies, including ours, have shown that TNBC with small local tumors and extensive regional lymph node involvement exhibit highly aggressive biological behavior (29, 30). In contrast, a relatively larger tumor size without LN involvement may be a surrogate for biologically indolent disease of distant metastasis, such as T3N0M0 breast cancer (31). It is plausible that eTNBC patients with different T and N stages may also benefit differently from BCT. Hence, this study will further conduct subgroup analysis by dividing the cohort into T1N0M0, T1N+M0, T2-4N0M0, and T2-4N+M0 groups.

## Materials and methods

To investigate a sufficiently large sample size, we used the Surveillance, Epidemiology, and End Results (SEER) database of the National Cancer Institute; this is an open-access resource for epidemiologic and survival analyses of various cancers that comprises 18 high-quality population-based cancer registries with a very high estimated completeness of reporting. And we used data from Shenzhen Second People’s Hospital (SZSPH) between January 2006 and January 2016. This study followed the Strengthening the Reporting of Observational Studies in Epidemiology (STROBE) reporting guideline for cohort studies. All data are anonymized and are therefore exempt from institutional review board review and informed consent requirements.

The SEER*Stat 8.3.6 software from the National Cancer Institute was used to identify patients eligible for the study based on the following inclusion criteria: female sex, diagnosis between 2010 and 2016 (and not later to ensure adequate follow-up), breast ductal carcinoma on pathology, unilateral breast cancer, stages I–III, TNBC, single primary site, and known age at diagnosis. For the cohort in SZSPH, other conditions were the same as above except that the enrollment year was 2006-2016. Patients registered in the SEER database were followed until death; those who died after the follow-up cutoff date were considered alive as of that date. Staging was verified according to the seventh edition of the American Joint Committee on Cancer staging system (2010). The pathological diagnosis was based on the primary site according to the International Classification of Diseases for Oncology, Third Edition.

Overall survival (OS) and breast cancer-specific survival (BCSS) were calculated using the Kaplan–Meier method, with significant differences between different groups assessed using the log-rank test. We used univariate and multivariable Cox proportional hazards models to calculate the hazard ratios (HRs) of clinicopathological factors with respect to OS and BCSS, as well as their corresponding 95% confidence intervals (CIs). A nomogram was constructed to predict the probability of survival using the variables that were found to be significant independent predictors of the same. Harrell’s C-index and receiver operating characteristic analysis were used to evaluate the discriminative ability of the nomogram. A calibration curve was used to compare the associations between the predicted probabilities and actual outcomes. To adjust the comparisons and avoid bias stemming from retrospective trials, propensity score analysis was performed using the “Matching” package of R. All statistical analyses were performed using SPSS (version 24.0; IBM Corp, Armonk, NY, USA) or R (version 3.4.0; Vienna, Austria; http://www.R-project.org). All statistical tests were two-sided, and a *P* value <0.05 was considered significant.

## Results

### Patient characteristics

**Figure 1:**
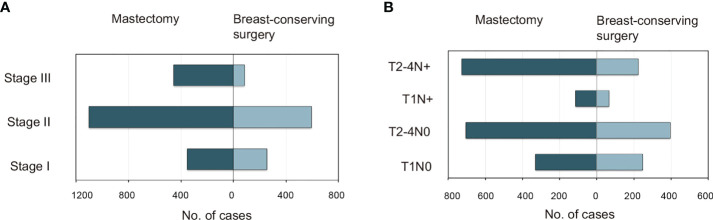
Distributions of patients <40 years with triple-negative breast cancer who underwent mastectomy versus breast-conserving surgery as categorized by **(A)** AJCC stage and **(B)** specific T and N substages. AJCC, American Joint Committee on Cancer.

Patient, disease, and treatment data are summarized in **Supplementary eTable 1** (**Supporting Materials**). The median (range) age at the diagnosis of TNBC was 35 (15–39) years, and the median (range) follow-up duration was 31 (0–83) months. A detailed flowchart of the patient selection process is shown in **Supplementary eFigure 1** (**Supporting Materials**). The study included 610 (21.4%), 1,695 (59.4%), and 549 (19.2%) patients with stage I, II, and III tumors, respectively. The 1-year, 3-year, and 5-year BCSS rates were 92.0%, 82.0%, and 79.0%, respectively, while the corresponding OS rates were 92.0%, 81.0%, and 78.0%, respectively. The majority of patients were 30–39 years of age; married; Caucasian; carrying health insurance; diagnosed with T1–2 stage, grade III, undifferentiated, invasive ductal carcinoma, ER-positive, and N0–N1 stage tumors; had undergone mastectomy; and had received chemotherapy (**Supplementary eTable 1** in the **Supporting Materials**). BCT and mastectomy were administered to 32.8% (934) and 67.2% (1,911) of the patients, respectively. Patients of any-stage eTNBC were more likely to undergo mastectomy (**Figure 1A**); the more advanced the TNM stage, the lower the frequency of BCT (**Figure 1A**). Among patients with the same T stage, those with lymph node (LN)-negative disease were more likely to undergo BCT than those with LN-positive disease (**Figure 1B**).

### Prognostic factors and treatment outcomes

Univariate Cox regression analysis revealed that unmarried status, African-American race, lack of health insurance, advanced T stage, advanced N stage, invasive lobular carcinoma (ILC) or mixed histologic type, undergoing BCT, and receiving radiotherapy were all significantly associated with poor BCSS and OS (**Table 1**). On multivariable Cox analysis, marital status, insurance status, T stage, and N stage were independent predictors of BCSS and OS (**Table 2**); moreover, histologic type was also a predictor of BCSS (**Table 2**). Married patients had better BCSS (adjusted HR, 0.78; *P*=0.038) and OS (adjusted HR, 0.78; *P*=0.03) than their divorced, separated, or widowed counterparts. Compared with uninsured patients, those who were insured had better BCSS (adjusted HR, 0.74; *P*=0.01) and OS (adjusted HR, 0.70; *P*=0.002). A more advanced T or N stage was associated with worse BCSS and OS. Patients with a mix of both invasive ductal carcinoma (IDC) and ILC had worse BCSS than did those with IDC alone (adjusted HR, 1.98; *P*=0.04). On univariate analysis, BCSS and OS were longer in patients who underwent mastectomy than in those who underwent BCT (HRs, 0.53 and 0.56, respectively; *P*<0.0001) (**Table 1**). However, these differences were not significant on multivariable analysis (adjusted HRs for BCSS and OS, 0.85 [*P*=0.23] and 0.81 [*P*=0.23], respectively) (**Table 2**). On univariate analysis, BCSS and OS were worse among patients who received radiotherapy than among those who did not (HRs, 1.46 and 1.53, respectively; *P*<0.0001) (**Table 1**), whereas this difference was not significant on multivariable analysis (adjusted HRs, 0.90 [*P*=0.41] and 0.87 [*P*=0.23], respectively) (**Table 2**).

**Table 1:** Univariate analysis of factors potentially predictive of overall survival and breast cancer-specific survival.

Variable	OS		BCSS	
	HR (95% CI)	*P* value	HR (95% CI)	*P* value
Age of diagnosis				
<30	Reference		Reference	
30-39	0.99 (0.73-1.34)	0.93	1.02 (0.74-1.40)	0.90
Marital status				
Not married^a^	Reference		Reference	
Married	0.66 (0.53-0.81)	<0.001	0.67 (0.54-0.83)	<0.0001
Unknown	0.78 (0.48-1.27)	0.32	0.79 (0.48-1.30)	0.36
Ethnic group				
Caucasian	Reference		Reference	
African-American	1.43 (1.13-1.80)	0.00	1.39 (1.01-1.77)	0.01
Other^b^	0.94 (0.65-1.36)	0.73	0.89 (0.61-1.31)	0.56
Unknown	0.00 (0.00-2.90E+81)	0.92	0.00 (0.00-8.95E+83)	0.92
Insurance				
Uninsured	Reference		Reference	
Insured	0.54 (0.44-0.67)	<0.001	0.574 (0.46-0.72)	<0.001
Unknown	0.24 (0.06-0.98)	0.05	0.266 (0.07-1.08)	0.06
AJCC T stage				
T1	Reference		Reference	
T2	1.79 (1.32-2.42)	<0.001	1.90 (1.38-2.60)	<0.001
T3	4.39 (3.13-6.16)	<0.001	4.38 (3.10-6.24)	<0.001
T4	9.37 (6.42-13.69)	<0.001	9.88 (6.68-14.62)	<0.001
AJCC N stage				
N0	Reference		Reference	
N1	3.04 (2.36-3.92)	<0.0001	3.05 (2.34-3.97)	<0.0001
N2	7.85 (5.76-10.70)	<0.0001	8.05 (5.86-11.08)	<0.0001
N3	10.58 (7.62-14.70)	<0.0001	11.14 (7.98-15.57)	<0.0001
Grade				
I	Reference		Reference	
II	1.97 (0.27-14.37)	0.50	1.88 (0.26-13.73)	0.63
III and undifferentiated	1.33 (0.19-9.44)	0.78	1.26 (0.18-8.95)	0.82
Unknown	1.65 (0.22-12.43)	0.63	1.55 (0.21-11.77)	0.67
Histologic type				
IDC	Reference		Reference	
ILC	6.69 (2.76-16.19)	<0.0001	5.67 (2.11-15.22)	0.00
Mixed IDC and ILC	3.75 (2.05-6.84)	<0.0001	3.97 (2.17-7.24)	<0.0001
Other type	0.76 (0.52-1.12)	0.16	0.78 (0.53-1.15)	0.22
Surgery				
Breast conservation	Reference		Reference	
Mastectomy	0.53 (0.42-0.68)	<0.0001	0.56 (0.43-0.72)	<0.0001
Chemotherapy				
No	Reference		Reference	
Yes	1.49 (0.95-2.33)	0.08	1.56 (0.97-2.51)	0.07
Radiation				
No	Reference		Reference	
Yes	1.46 (1.19-1.79)	<0.0001	1.53 (1.24-1.89)	<0.0001

AJCC, American Joint Committee on Cancer; BCSS, breast cancer-specific survival; CI, confidence interval; HR, hazard ratio; IDC, invasive ductal carcinoma; ILC, invasive lobular carcinoma; OS, overall survival.

a Includes divorced, separated, single (never married), and widowed.

**Table 2:** Multivariable analysis of factors potentially predictive of overall survival and breast cancer-specific survival.

Variable	OS		BCSS	
	HR (95% CI)	*P* value	HR (95% CI)	*P* value
Age of diagnosis				
<30	Reference		Reference	
30-39	1.32 (0.96-1.80)	0.09	1.35 (0.98-1.87)	0.07
Marital status				
Not married^a^	Reference		Reference	
Married	0.78 (0.62-0.98)	0.03	0.78 (0.61-0.99)	0.04
Unknown	0.96 (0.58-1.59)	0.87	0.96 (0.57-1.61)	0.87
Ethnic group				
Caucasian	Reference		Reference	
African-American	1.08 (0.84-1.38)	0.56	1.05 (0.81-1.35)	0.73
Other^b^	1.03 (0.71-1.50)	0.87	0.97 (0.66-1.43)	0.87
Unknown	0 (0-1.57E+70)	0.92		0.92
Insurance				
Uninsured	Reference		Reference	
Insured	0.70 (0.56-0.88)	0.00	0.74 (0.58-0.94)	0.01
Unknown	0.36 (0.09-1.45)	0.15	0.39 (0.09-1.57)	0.18
AJCC T stage				
T1	Reference		Reference	
T2	1.51 (1.11-2.05)	0.01	1.60 (1.16-2.20)	0.00
T3	2.42 (1.70-3.47)	<0.001	2.42 (1.67-3.52)	<0.001
T4	4.01 (2.66-6.03)	<0.001	4.26 (2.80-6.50)	<0.001
AJCC N stage				
N0	Reference		Reference	
N1	2.66 (2.04-3.48)	<0.001	2.65 (2.01-3.50)	<0.001
N2	6.07 (4.34-8.49)	<0.001	6.22 (4.40-8.78)	<0.001
N3	7.48 (5.17-10.82)	<0.001	7.96 (5.46-11.59)	<0.001
Grade				
I	Reference		Reference	
II	0.78 (0.11-5.76)	0.81	0.71 (0.10-5.26)	0.74
III and undifferentiated	0.55 (0.08-3.97)	0.55	0.50 (0.07-3.60)	0.49
Unknown	0.56 (0.03-4.34)	0.58	0.51 (0.07-3.95)	0.52
Histologic type				
IDC	Reference		Reference	
ILC	0.92 (0.34-2.46)	0.86	0.74 (0.25-2.18)	0.58
Mixed IDC and ILC	1.91 (0.99-3.68)	0.05	1.98 (1.03-3.83)	0.04
Other type	0.76 (0.51-1.12)	0.16	0.78 (0.52-1.16)	0.21
Surgery				
Breast conservation	Reference		Reference	
Mastectomy	0.81 (0.69-1.10)	0.23	0.85 (0.65-1.11)	0.23
Chemotherapy				
No	Reference		Reference	
Yes	1.00 (0.63-1.61)	0.99	1.04 (0.63-1.70)	0.89
Radiation				
No	Reference		Reference	
Yes	0.87 (0.69-1.10)	0.23	0.90 (0.71-1.15)	0.41

AJCC, American Joint Committee on Cancer; BCSS, breast cancer-specific survival; CI, confidence interval; HR, hazard ratio; IDC, invasive ductal carcinoma; ILC, invasive lobular carcinoma; OS, overall survival.

a Includes divorced, separated, single (never married), and widowed.

### Subgroup analysis stratified by TNM stage

**Figure 2:**
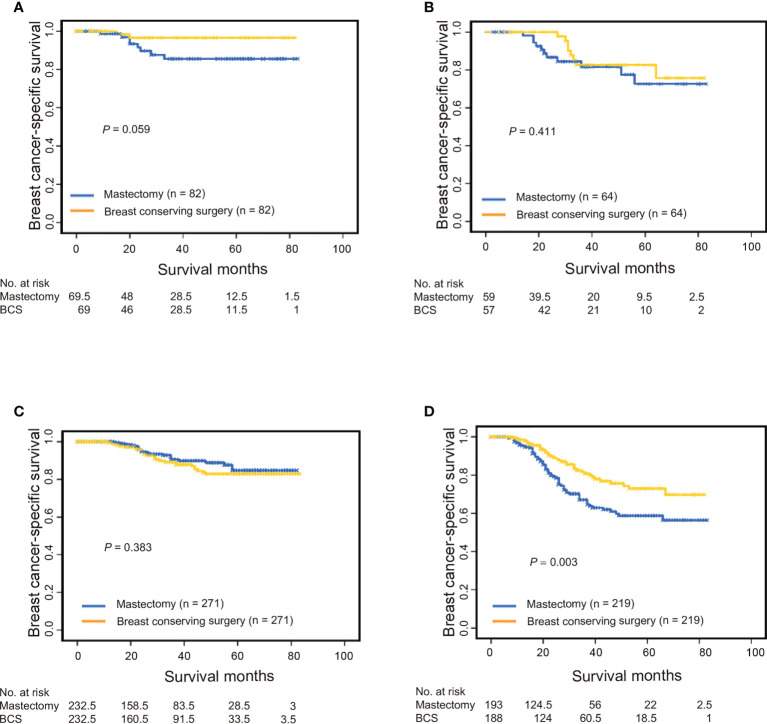
Breast cancer-specific survival among propensity score-matched patients with triple-negative breast cancer who underwent breast conservation surgery versus mastectomy. **(A)** Patients with T1N0M0 stage, **(B)** patients with T1N+M0 stage, **(C)** patients with T2-4N0M0 stage, and **(D)** patients with T2-4N+M0 stage. BCS, breast-conserving surgery.

T and N stages were found to be strong indicators of both BCSS and OS. To avoid potential biases while evaluating the association between surgical procedure type and TNM stage, we performed a stratification analysis of patients treated with BCT versus mastectomy based on their different T and N stages. BCT was associated with longer BCSS and OS than was mastectomy in the T1N0M0 and T2-4N+M0 subgroups; however, there were no such differences for the T1N+M0 or T2-4N0M0 groups (**Figure 2** and **Supplementary eFigure 2** in the **Supporting Materials**). To ensure that differences in outcomes were not attributed to baseline variations in demographic and clinical characteristics, we performed a 1:1 (BCT: mastectomy) propensity score-matched case-control analysis (579 patients with T1N0M0 tumors, 128 with T1N+M0 tumors, 542 with T2-4N0M0 tumors, and 952 with T2-4N+M0 tumors); the results concurred with pre-matching findings (**Supplementary eFigure 3** and **Supplementary eFigure 4** in the **Supporting Materials**).

### Prognostic nomogram for patients with eTNBC

**Figure 3:**
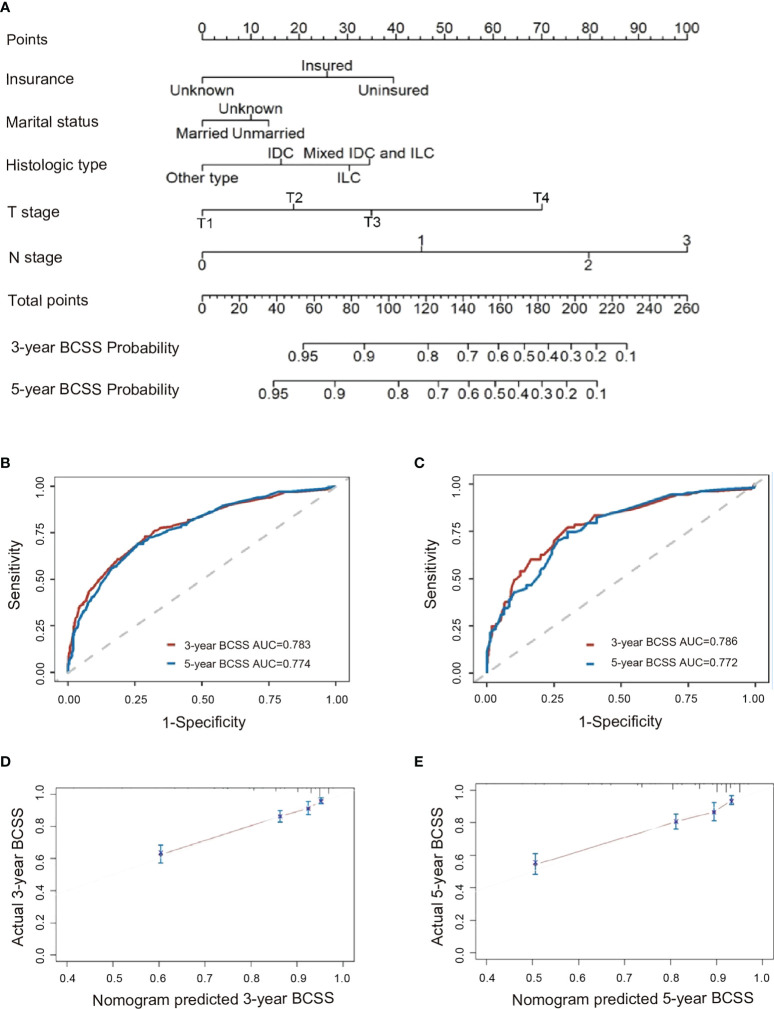
Analysis of factors predictive of BCSS among patients <40 years with triple-negative breast cancer. **(A)** Nomogram for predicting 3-year and 5-year BCSS. Also shown are ROC curves for the **(B)** training set and **(C)** validation set, as well as the nomogram’s calibration curves in terms of **(D)** 3-year BCSS and **(E)** 5-year BCSS. AUC, area under the curve; BCSS, breast cancer-specific survival; ROC, receiver operating characteristic.

Per modeling requirements, 2,854 patients were randomly assigned to the training cohort (n=1,998) and validation cohort (n=856); there were no significant differences in their baseline clinicopathologic characteristics except for age and histological type (**Supplementary eTable 2** in the **Supporting Materials**). Using the training set, a nomogram was constructed by incorporating factors found to be associated with BCSS per the Cox proportional hazards regression model (**Figure 3A**). N stage had the greatest influence (maximum of 100 points), followed by T stage (70 points), insurance status (40 points), histologic type (35 points), tumor size (24 points), and marital status (13 points). In the training cohort (internal validation) and validation cohort (external validation), the C-indices for BCSS prediction in the nomogram were 0.774 (95% CI, 0.741–0.807) and 0.768 (95% CI, 0.718–0.818), respectively. We determined the reliability of the nomogram using receiver operating characteristic analysis (**Figures 3B, C**); the areas under the curve for predicting the 3-year and 5-year BCSS rates were 0.783 and 0.774, respectively, in the training set and 0.786 and 0.772, respectively, in the validation set. Moreover, the calibration plots showed consistency between the nomogram-predicted and actual 3-year BCSS (**Figure 3D**) and 5-year BCSS (**Figure 3E**).

### Nomogram validation in SZSPH cohort

**Figure 4:**
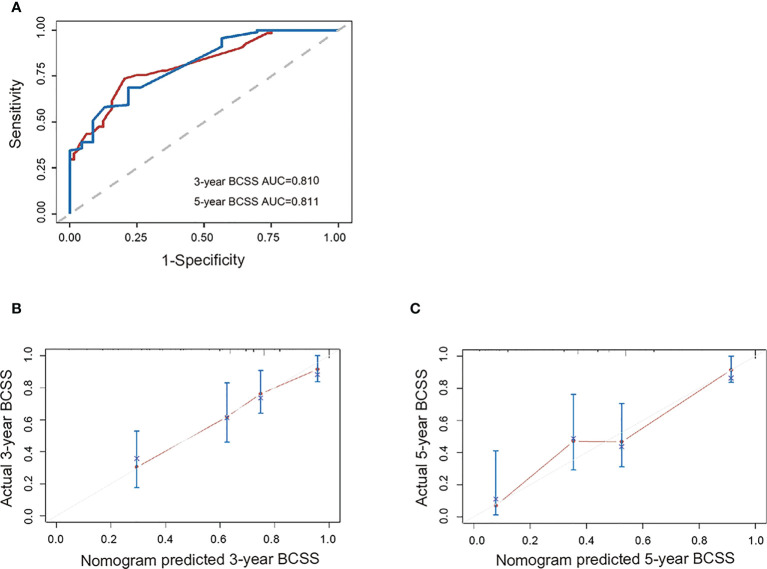
External validation of the nomogram. **(A)** ROC curve for the external validation set, as well as the nomogram’s calibration curves in terms of **(B)** 3-year BCSS and **(C)** 5-year BCSS. AUC, area under the curve; BCSS, breast cancer-specific survival; ROC, receiver operating characteristic.

A total of 214 patients were enrolled in the external validation cohort. Nomogram validation was further processed in the dataset of our center. Patient, disease, and treatment data are summarized in **Supplementary eTable 3** (**Supporting Materials**). The median (range) age at the diagnosis of TNBC was 33(16–39) years, and the median (range) follow-up duration was 26 (0–83) months. The Harrell’s C-index for the nomograms for predicting BCSS were 0.723. The ROC curves for predicting 3- and 5-year BCSS in our cohorts were presented in **Figure 4A**. The calibration curves were shown in **Figures 4B, C** which demonstrated a well agreement between the actual and nomogram-predicted survival rates. These results demonstrated that the nomograms were useful tools for the prediction of survival in patients with eTNBC.

## Discussion

In this study, patients of any-stage eTNBC were more likely to undergo mastectomy. Not married, uninsured, more advanced T stage and N stage were independent predictors of poor BCSS and OS. Moreover, the histologic type of mixed IDC and ILC was also a predictor of poor BCSS. Subgroup analysis utilizing propensity score-matching method showed that BCT was associated with longer BCSS and OS than was mastectomy in the T1N0M0 and T2-4N+M0 subgroups; however, there were no such differences for the T1N+M0 or T2-4N0M0 groups. The nomograms for predicting the 3-year and 5-year BCSS rates were constructed and proved to be quite reliable and robust.

Because eTNBC constitutes a small fraction of diagnosed breast cancers, it has been challenging to definitively characterize its prognostic factors, biological behavior, and response to multimodality therapy. Furthermore, the recommended surgical modalities proposed for TNBC remain controversial (16, 18, 19, 21–23). When compared to published data from older counterparts, a higher proportion of young patients with TNBC in our study were African-American, had histologic types other than IDC and ILC, had grade III and undifferentiated diseases, presented with more advanced T and N stage, and had received chemotherapy (32). Furthermore, both T stage and N stage are strongly associated with prognosis regardless of age (32). However, compared to patients with TNBC overall (32, 33), the prognoses of those who were young were not associated with tumor grade, chemotherapy, or radiotherapy according to our multivariable Cox analysis, whereas marital and health insurance statuses were. Unmarried women are reportedly more likely to develop advanced-stage breast cancer, as having a spouse may represent a factor supporting the early detection of malignancy (34, 35). Social support from a partner might also influence treatment compliance, particularly among women experiencing side effects (36). Previous studies also showed that uninsured premenopausal patients are more likely to forgo effective treatment for financial reasons and to opt out of follow-up programs, causing recurrences to remain under-detected (36, 37).

According to SEER data, the rate of BCT between 2000 and 2006 in the United States was 55–60% (38). Data from more than 20 breast cancer treatment centers certified by the European Society of Breast Cancer Specialists showed that their BCT rates between 2003 and 2010 ranged between 75% and 80% (39). In our study, the BCT rate among eTNBC women was lower than that in patients overall. Possible reasons for this include: (i) Young age is an independent risk factor for ipsilateral breast tumor recurrence after BCT (40, 41), and some surgeons or patients might therefore choose mastectomy instead. (ii) While molecular typing does not dictate BCT eligibility, patients with non-luminal A-type tumors may carry an increased risk of local recurrence afterwards (41–44). Although standardized systemic therapy reduces this risk (41–44), patients with TNBC who are candidates for BCT may opt for mastectomy instead (45). (iii) Since our study included more patients with advanced T stage, the proportion of those eligible for BCT was lower. (iv) The N stage and histologic grade of TNBCs in young patients were more advanced, leading to a greater risk of ipsilateral breast tumor recurrence (41) and higher mastectomy rate. (v) There was a greater proportion of African-American patients in the eTNBC cohort, the majority of whom were not candidates for BCT (46).

Whether locoregional treatment of eTNBC influences long-term prognosis remains poorly understood. Previous studies suggested that in TNBC, BCT achieved better locoregional recurrence-free, disease-free, and OS rates compared with mastectomy, and age was not an independent prognostic factor (18, 21, 47). While our results found no adverse effect of BCT on BCSS or OS in patients with eTNBC compared with mastectomy. In order to identify which subgroup of patients would benefit from BCT, we further conducted subgroup analysis. There is evidence, including from our previous study, that a very small tumor size is indicative of a biologically aggressive disease in patients with extensive LN involvement (29, 30). In contrast, tumors that fail to metastasize to regional LNs, even at a late stage (e.g., T3N0 tumors), may reflect a more biologically indolent phenotype (31). Therefore, we divided our cohort into T1N0M0, T1N+M0, T2-4N0M0, and T2-4N+M0 stages for subgroup analysis. On the one hand, we found that BCT was associated with longer BCSS and OS than was mastectomy in the T1N0M0 and T2-4N+M0 subgroups. There are several possible reasons. Although surgery does provide superior local control within the resected tissue, the treated volume is larger with tangential radiotherapy than with simple mastectomy and typically encompasses the entire breast tissue as well as skin, the subcutaneous lymphatic plexus, part of the muscle, and regional lymphatics (48). Incidental irradiation may also eliminate microscopic disease outside the breast tissue (49). Moreover, postoperative radiotherapy not only reduces local recurrence but also diminishes the risk of distant relapse owing to mechanisms such as the abscopal effect (50, 51). Preventing locoregional recurrence reduces the risk of breast cancer-specific death and may improve OS (52). Lastly, patients eligible for BCT who undergo total mastectomy instead may not be followed as closely as those who undergo BCT; hence, recurrence and metastasis may not be detected and managed in a timely manner. On the other hand, we found that patients with T1N+M0 and T2-4N0M0 tumors who underwent BCT had similar BCSS and OS rates as those who underwent mastectomy. As for those with T1N+M0 (a potentially aggressive disease) (29, 30), improved local treatment was not sufficient to produce survival benefits, and more intensive systemic treatment was required. For those with biologically indolent T2-4N0M0 tumors (31), mastectomy seemed to be sufficient for local control and survival; thus BCT did not appear to improve any more survival benefit. Taken together, BCT is associated with a prognosis similar to that of mastectomy among eTNBC patients but produces more favorable outcomes among those with T1N0M0 and T2-4N+M0 tumors. Therefore, BCT could be a preferable option for eTNBC patients.

Given the unique clinicopathological and social image-related features of eTNBC, we used our data to establish a reliable nomogram for predicting BCSS. Because the risk of distant TNBC recurrence typically peaks 1–3 years after diagnosis and declines rapidly thereafter (10), the nomogram was devised to calculate the 3- and 5-year BCSS rates. The calibration curves, receiver operating characteristic analysis, and C-indices of both internal and external validation demonstrated that it performs satisfactorily and can therefore be used for personalized risk prediction and treatment guidance for eTNBC patients.

This study had several strengths. First, we investigated the prognoses of patients with various combinations of T and N stages. Second, we included a larger cohort of patients than did other studies despite the low incidence of eTNBC and evaluated the association between surgical approaches and OS and BCSS in these patients for the first time. The BCSS data were derived from the SEER database (a population-based registry) and not a single-hospital database. However, this study also had some limitations. First, the retrospective nature of our study may also have introduced a certain level of bias. Second, potential limitation of SEER dataset also included unrecorded factors, underreported and inadequate adjuvant therapy data, differences in coding and reporting, and patient migration between SEER registry locations. Finally, breast cancer diagnosed in patients under the age of 40 years is more likely to have hereditary causes, which may be related to a slightly higher risk of recurrence and death. However, because of the lack of genetic data in the SEER datasets, we could not incorporate this factor into our nomograms; this may have led to some predictive bias.

Generally, early age of breast cancer onset is considered an indicator of cancer susceptibility genes, typically BRCA1 or BRCA2 (53). BRCA mutations are the most common genetic variabilities in breast cancer and closely associated with aggressive clinical and biologic course of breast cancer, especially the TNBC subtype. It is reported that the prevalence of BRCA mutation in eTNBC was around 18.87% (54, 55). Although the risk for recurrent breast cancer or contralateral breast cancer was higher in BRCA mutated TNBC, advances in biology and systemic therapy have decreased the risk to an acceptable level (56, 57). Simultaneously, the same tendency was revealed in patients with BRCA mutated eTNBC, no matter what surgical procedures were performed (55). It is therefore rational to propose that predictive bias caused by not incorporating genetic data into our nomograms were acceptable.

In conclusion, our findings indicate that a localized surgical approach might be preferable for patients with eTNBC who have certain clinicopathological features (namely T1N0M0- and T2-4N+M0-stage tumors). Marital status, health insurance status, T stage, N stage, and histological type were independent prognostic factors, and a nomogram established based on these variables successfully predicted the 3- and 5-year survival probabilities among these patients.

## Data availability statement

Publicly available datasets were analyzed in this study. This data can be found here: http://www.seer.cancer.gov/seerstat.

## Ethics statement

We obtained permission to access the SEER research data files using the reference number 15223-Nov2019. The research protocol was approved by the Ethics Committee of shenzhen Second People's Hospital, Shenzhen University, Shenzhen, Guangdong, China. Because The data released by the SEER database are anonymized and patients cannot be identified, Ethics Committee of Shenzhen Second People's Hospital exempted this study from review and informed consent requirements (Document No: 2022017). The methods were performed in accordance with the principles stated in the Declaration of Helsinki.

## Author contributions

Y-ZZ: conceptualization, data curation, formal analysis, methodology, software-based analysis, writing of the original draft, and funding acquisition. YL: data curation and formal analysis. Z-HD: review and editing of the manuscript. G-WL: validation. NX: conceptualization, project administration, and supervision. All authors contributed to the article and approved the submitted version.

## Funding

The design and conduct of the study were supported by the National Natural Science Foundation of China (Grant Numbers: 81902682, 82273397). Collection, management, analysis, and interpretation of the data was supported by Guangdong Basic and Applied Basic Research Foundation (Grant Number: 2021A1515011122). Preparation, review and submission of the manuscript was supported by Shenzhen Science and Technology Project (Grant Number: JCYJ20210324103003010) and Clinical Research Project of Shenzhen Second People’s Hospital (Grant Number: 20223357015).

## Acknowledgments

Y-ZZ would like to thank her parents, husband and daughter Jelly Deng for their strong moral support during her difficult time.

## Conflict of interest

The authors declare that the research was conducted in the absence of any commercial or financial relationships that could be construed as a potential conflict of interest.

## Publisher’s note

All claims expressed in this article are solely those of the authors and do not necessarily represent those of their affiliated organizations, or those of the publisher, the editors and the reviewers. Any product that may be evaluated in this article, or claim that may be made by its manufacturer, is not guaranteed or endorsed by the publisher.
