# Supplementary material for: Determining prognostic factors and optimal surgical intervention for early-onset triple-negative breast cancer
Source: Front Oncol. 2022 Oct 28;12:910765. doi: 10.3389/fonc.2022.910765 (PMC9650239; doi:10.3389/fonc.2022.910765)
Supplement: Supplementary file 1 [file DataSheet_1.docx]

Supplementary Material


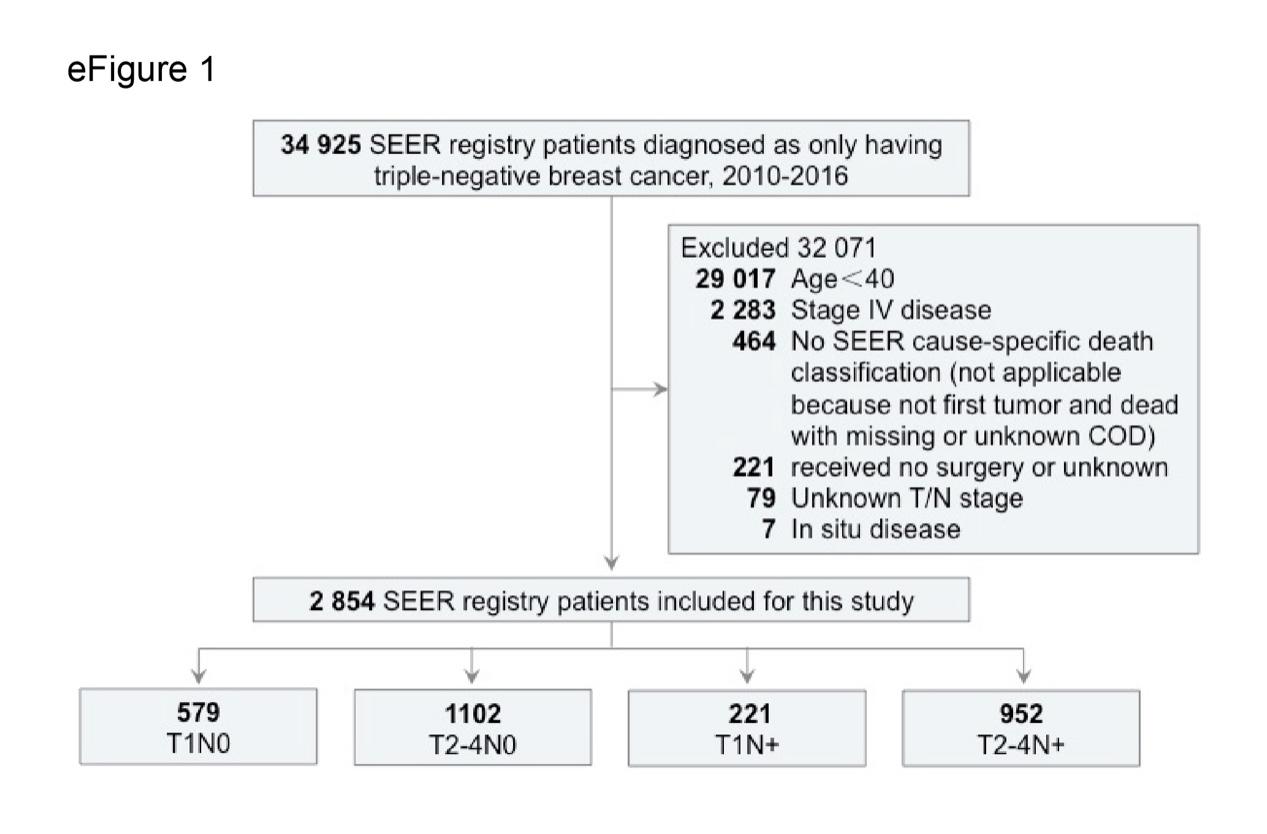


**Supplementary eFigure 1.** Flowchart of the case selection process in the study.


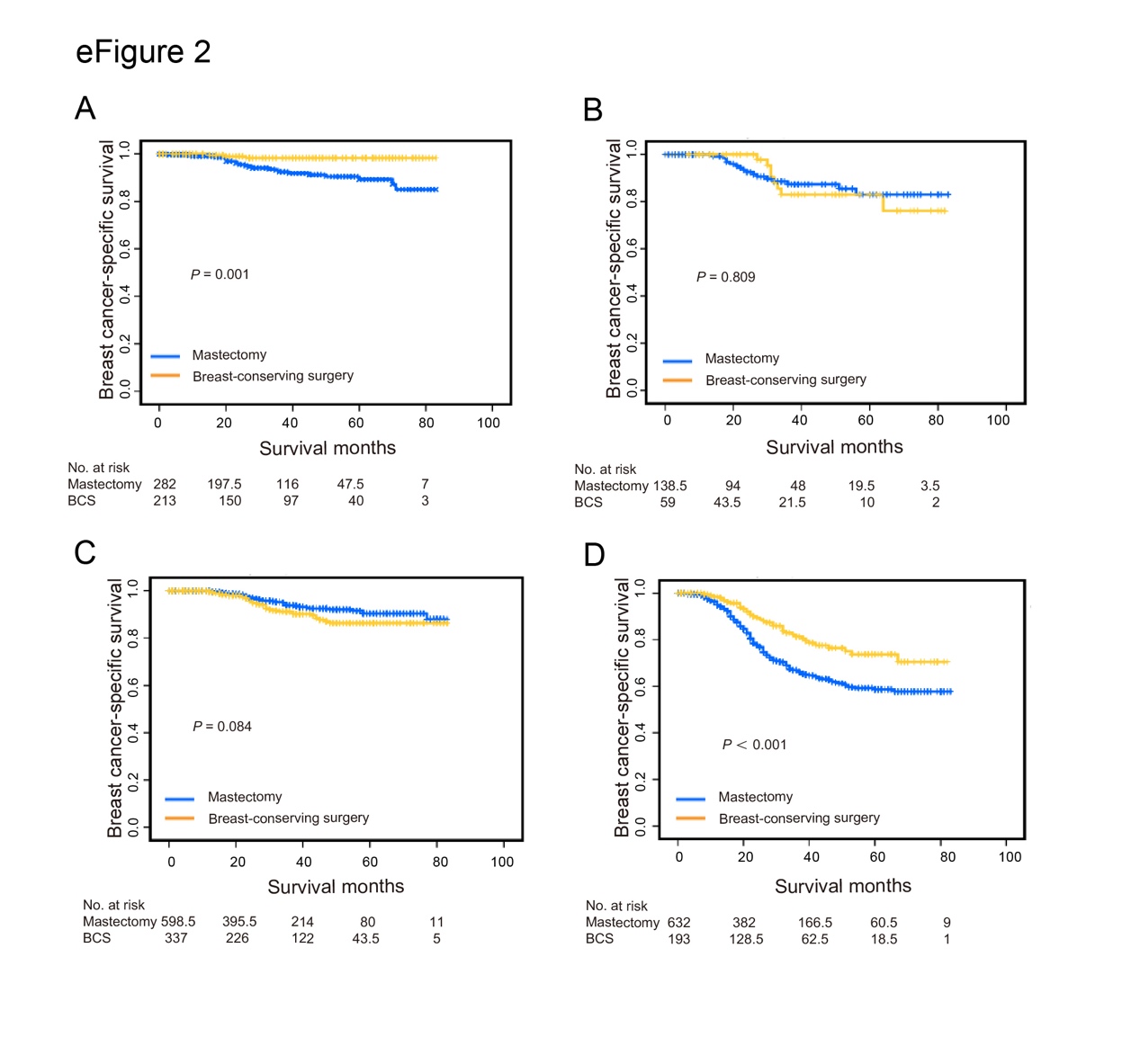


**Supplementary eFigure 2**: Breast cancer-specific survival among patients who underwent breast conservation surgery versus mastectomy. (**A**) Patients with T1N0M0 stage, (**B**) patients with T1N+M0 stage, (**C**) patients with T2-4N0M0 stage, and (**D**) patients with T2-4N+M0 stage. BSC, breast conserving surgery.


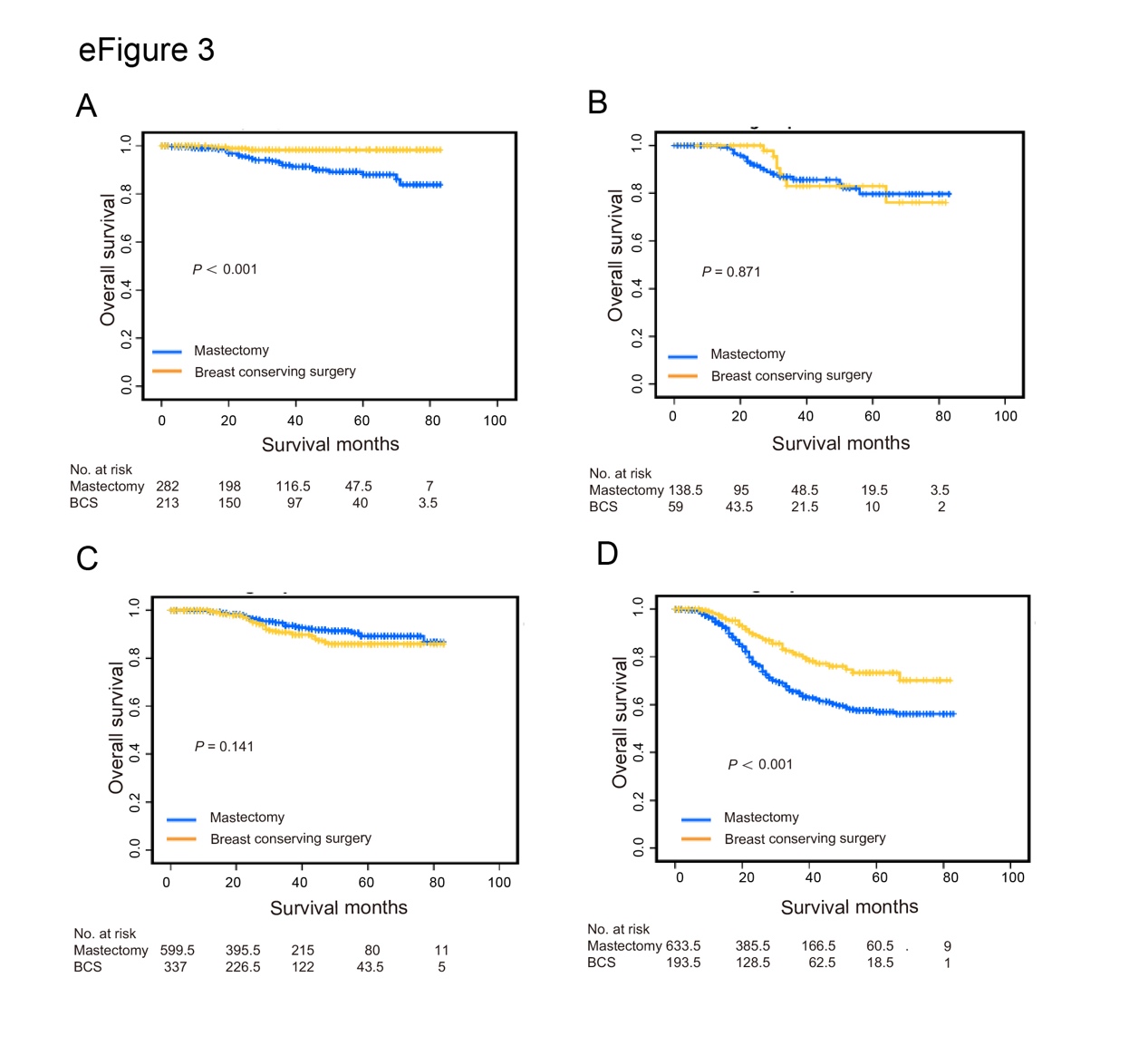


**Supplementary eFigure 3**: Overall survival among patients who underwent breast conservation surgery versus mastectomy. (**A**) Patients with T1N0M0 stage, (**B**) patients with T1N+M0 stage, (**C**) patients with T2-4N0M0 stage, and (**D**) patients with T2-4N+M0 stage. BCS, breast-conserving surgery.


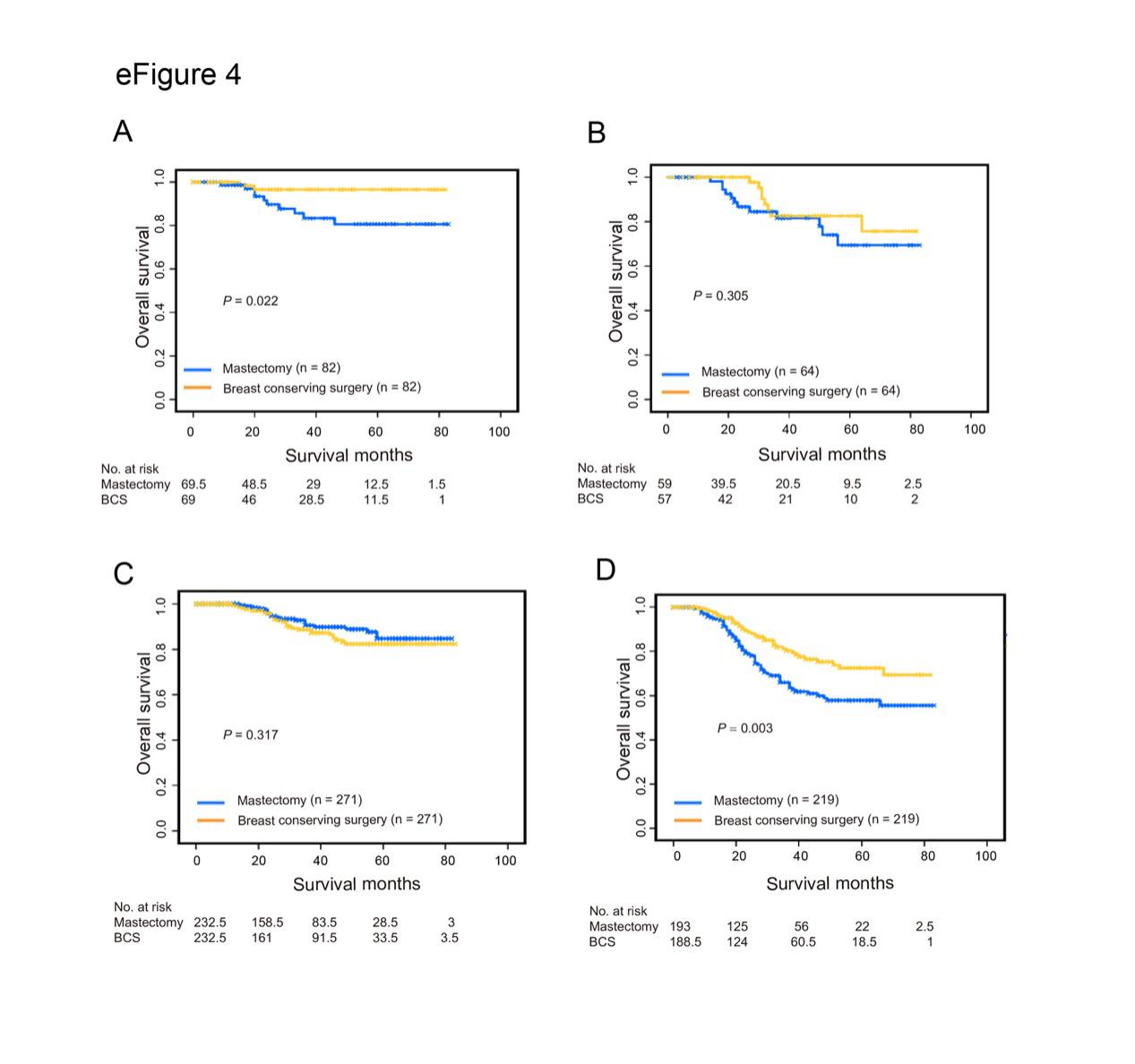


**Supplementary eFigure 4**: Overall survival among propensity score-matched patients who underwent breast conservation surgery versus mastectomy. (**A**) Patients with T1N0M0 stage, (**B**) patients with T1N+M0 stage, (**C**) patients with T2-4N0M0 stage, and (**D**) patients with T2-4N+M0 stage. BCS, breast-conserving surgery.

| **Table S1. Characteristics of women with early-onset triple-negative breast cancer in the SEER resistry, N = 2854** | | | | |
| --- | --- | --- | --- | --- |
|  |  |  |  |  |
|  |  | Surgical approaches | |  |
| Characteristics | Total | Mastectomy | BCS | *P* value |
| Age of diagnosis |  |  |  |  |
| ＜30 | 374 (13.1) | 274 (73.3) | 100 (26.7) | 0.01 |
| 30-39 | 2480 (86.9) | 1646 (66.4) | 834 (33.6) |  |
| Marital status |  |  |  |  |
| Married | 1582 (55.4) | 1094 (69.2) | 488 (30.8) | 0.05 |
| Not married^a^ | 1140 (39.9) | 744 (65.1) | 398 (34.9) |  |
| Unknown | 130 (4.6) | 82 (63.1) | 48 (36.9) |  |
| Ethnic group |  |  |  |  |
| Caucasian | 1965 (68.9) | 1364 (69.4) | 601 (30.6) | 0.00 |
| African-American | 594 (20.8) | 382 (64.3) | 212 (35.7) |  |
| Other^b^ | 273 (9.6) | 161 (59.0) | 112 (41.0) |  |
| Unknown | 22 (0.8) | 13 (59.1) | 9 (40.9) |  |
| Insurance |  |  |  |  |
| Insured | 2105 (73.8) | 1425 (67.7) | 680 (32.3) | 0.72 |
| Uninsured | 38 (1.3) | 470 (66.1) | 241 (33.9) |  |
| Unknown | 711 (24.9) | 25 (65.8)) | 13 (34.3) |  |
| AJCC T stage |  |  |  |  |
| T1 | 800 (28.0) | 486 (60.8) | 314 (39.3) | ＜0.001 |
| T2 | 1559 (54.6) | 1010 (64.8) | 549 (35.2) |  |
| T3 | 354 (12.4) | 298 (84.2) | 56 (15.8) |  |
| T4 | 141 (4.9) | 126 (89.4) | 15 (10.6) |  |
| AJCC N stage |  |  |  |  |
| N0 | 1680 (58.9) | 1037 (61.7) | 644 (38.3) | ＜0.001 |
| N1 | 849 (29.8) | 613 (72.2) | 236 (27.8) |  |
| N2 | 185 (6.5) | 151 (81.6) | 34 (18.4) |  |
| N3 | 139 (4.9) | 119 (85.6) | 20 (14.4) |  |
| Grade |  |  |  |  |
| I | 11 (0.4) | 10 (90.9)) | 1 (9.1)) | 0.07 |
| II | 239 (8.4) | 175 (73.2) | 64 (26.8) |  |
| III and undifferentiated | 2494 (87.4) | 1662 (66.6) | 832 (33.4) |  |
| Unknown | 110 (3.9) | 73 (66.4) | 37 (33.6) |  |
| Histologic type |  |  |  |  |
| IDC | 2524 (88.4) | 1700 (67.4) | 824 (32.6) | 0.00 |
| ILC | 8 (0.3) | 8 (100.0) | 0 (0.0) |  |
| Mixed IDC and ILC | 32 (1.1) | 29 (90.6) | 3 (9.4) |  |
| Other type | 290 (10.2) | 183 (63.1) | 107 (36.9) |  |
| Chemotherapy |  |  |  |  |
| No | 204 (7.15) | 119 (58.3) | 85 (41.7) | 0.01 |
| Yes | 2650 (92.85) | 1801 (68.0) | 849 (32.0) |  |
| Radiation |  |  |  |  |
| No | 1477 (51.75) | 1212 (82.1) | 265 (17.9) | ＜0.001 |
| Yes | 1377 (48.25) | 708 (51.4) | 669 (48.6) |  |
| Abbreviations: AJCC, American Joint Committee on Cancer; BCS, breast-conserving surgery; ER, estrogen receptor; IDC, invasive ductal carcinoma; ILC, invasive lobular carcinoma; PR, progesterone receptor. ^a^Includes divorced, separated, single (never married), and widowed.  ^b^American Indian/Alaskan native, or Asian/Pacific Islander. | | | | |
|  |  |  |  |  |
|  |  |  |  |  |
|  |  |  |  |  |
|  |  |  |  |  |

| **Table S2. Characteristics of the patients in the SEER registry** | | | | | |
| --- | --- | --- | --- | --- | --- |
| Characteristics | Training set | | Validating set | | ^a^*P* value |
|  | No. of patients | % | No. of patients | % |  |
| Age of diagnosis |  |  |  |  | 0.03 |
| ＜30 | 244 | 12.21 | 130 | 15.19 |  |
| 30-39 | 1754 | 87.79 | 726 | 84.81 |  |
| Marital status |  |  |  |  | 0.28 |
| Married | 1115 | 55.81 | 467 | 54.56 |  |
| Not married^b^ | 800 | 40.04 | 342 | 39.95 |  |
| Unknown | 83 | 4.15 | 47 | 5.49 |  |
| Ethnic group |  |  |  |  | 0.49 |
| Caucasian | 1387 | 69.42 | 578 | 67.52 |  |
| African-American | 404 | 20.22 | 190 | 22.20 |  |
| Other^c^ | 191 | 9.56 | 82 | 9.58 |  |
| Insurance |  |  |  |  | 0.53 |
| Insured | 1464 | 73.27 | 641 | 74.88 |  |
| Uninsured | 505 | 25.28 | 206 | 24.07 |  |
| Unknown | 29 | 1.45 | 9 | 1.05 |  |
| AJCC T stage |  |  |  |  | 0.75 |
| T1 | 559 | 27.98 | 241 | 28.15 |  |
| T2 | 1083 | 54.20 | 476 | 55.61 |  |
| T3 | 253 | 12.66 | 101 | 11.80 |  |
| T4 | 103 | 5.16 | 38 | 4.44 |  |
| AJCC N stage |  |  |  |  | 0.51 |
| N0 | 1171 | 58.61 | 510 | 59.58 |  |
| N1 | 608 | 30.43 | 241 | 28.15 |  |
| N2 | 123 | 6.16 | 62 | 7.24 |  |
| N3 | 96 | 4.80 | 43 | 5.02 |  |
| Grade |  |  |  |  | 0.74 |
| I | 7 | 0.35 | 4 | 0.47 |  |
| II | 163 | 8.16 | 76 | 8.88 |  |
| III and  undifferentiated | 1749 | 87.54 | 745 | 87.03 |  |
| Histologic type |  |  |  |  | 0.01 |
| IDC | 1761 | 88.14 | 763 | 89.14 |  |
| ILC | 6 | 0.30 | 2 | 0.23 |  |
| Mixed IDC and ILC | 15 | 0.75 | 17 | 1.99 |  |
| Other type | 216 | 10.81 | 74 | 8.64 |  |
| Surgery |  |  |  |  | 0.38 |
| Breast conservation | 664 | 33.23 | 270 | 31.54 |  |
| Mastectomy | 1334 | 66.77 | 586 | 68.46 |  |
| Chemotherapy |  |  |  |  | 0.16 |
| No | 134 | 6.71 | 70 | 8.18 |  |
| Yes | 1864 | 93.29 | 786 | 91.82 |  |
| Radiation |  |  |  |  | 0.14 |
| No | 1016 | 50.85 | 461 | 53.86 |  |
| Yes | 982 | 49.15 | 395 | 46.14 |  |
| Abbreviations: AJCC, American Joint Committee on Cancer; ER, estrogen receptor; IDC, invasive ductal carcinoma; ILC, invasive lobular carcinoma; PR, progesterone receptor. ^a^P values were calculated by chi-square test. ^b^Includes divorced, separated, single (never married), and widowed.  ^c^American Indian/Alaskan native, or Asian/Pacific Islander. | | | | | |

| **Table S3.** Characteristics of women with early-onset triple-negative breast cancer in our center, N = 214 | | |  |
| --- | --- | --- | --- |
|  |  |  |  |
| Characteristics | Number of patients | Proportion of total (%) |  |
| Age of diagnosis |  |  |  |
| ＜30 | 17 | 7.94 |  |
| 30-39 | 197 | 92.06 |  |
| Marital status |  |  |  |
| Married | 122 | 57.01 |  |
| Not married^a^ | 81 | 37.85 |  |
| Unknown | 11 | 5.14 |  |
| Insurance |  |  |  |
| Insured | 168 | 78.50 |  |
| Uninsured | 44 | 20.56 |  |
| Unknown | 2 | 0.93 |  |
| AJCC T stage |  |  |  |
| T1 | 77 | 35.98 |  |
| T2 | 85 | 39.72 |  |
| T3 | 32 | 14.95 |  |
| T4 | 20 | 9.35 |  |
| AJCC N stage |  |  |  |
| N0 | 39 | 18.22 |  |
| N1 | 120 | 56.07 |  |
| N2 | 23 | 10.75 |  |
| N3 | 32 | 14.95 |  |
| Grade |  |  |  |
| I | 0 | 0.00 |  |
| II | 28 | 13.08 |  |
| III and undifferentiated | 186 | 86.92 |  |
| Histologic type |  |  |  |
| IDC | 195 | 91.12 |  |
| ILC | 3 | 1.40 |  |
| Mixed IDC and ILC | 6 | 2.80 |  |
| Other type | 10 | 4.67 |  |
| Surgery |  |  |  |
| Breast conservation | 64 | 29.91 |  |
| Mastectomy | 150 | 70.09 |  |
| Chemotherapy |  |  |  |
| No | 24 | 11.21 |  |
| Yes | 190 | 88.79 |  |
| Radiation |  |  |  |
| No | 98 | 45.79 |  |
| Yes | 116 | 54.21 |  |
| Abbreviations: AJCC, American Joint Committee on Cancer; ER, estrogen receptor; IDC, invasive ductal carcinoma; ILC, invasive lobular carcinoma; PR, progesterone receptor. ^a^Includes divorced, separated, single (never married), and widowed. | | |  |
|  |  |  |  |
|  |  |  |  |
|  |  |  |  |
|  |  |  |  |
|  |  |  |  |
